# Concurrent Particulate Matter and Heat Exposure in Working and Non-working Women in Rural Guatemala

**DOI:** 10.3390/atmos15101175

**Published:** 2024-09-30

**Authors:** Jaime Butler-Dawson, Grant Erlandson, Diana Jaramillo, Laura Calvimontes, Daniel Pilloni, James Seidel, Colton Castro, Karely Villarreal Hernandez, Lyndsay Krisher, Stephen Brindley, Miranda Dally, Alex Cruz, Katherine A James, Lee S Newman, Joshua Schaeffer, John L Adgate

**Affiliations:** 1 Department of Environmental and Occupational Health, Colorado School of Public Health, University of Colorado Anschutz Campus, Aurora, Colorado, USA; 2 Center for Health Work and Environment, Colorado School of Public Health, University of Colorado Anschutz Campus, Aurora, Colorado, USA; 3 Department of Environmental and Radiological Health Sciences, Colorado State University, Fort Collins, CO, USA; 4 Grupo Pantaleon, Guatemala City, Guatemala; 5 Department of Epidemiology, Colorado School of Public Health, University of Colorado Anschutz Campus, Aurora, Colorado, USA; 6 Division of Pulmonary Sciences and Critical Care Medicine, Department of Medicine, School of Medicine, University of Colorado Anschutz Campus, Aurora, Colorado, USA

**Keywords:** Environmental Exposures, Particulate Matter, Heat, Women, Agricultural Workers

## Abstract

High temperatures and air pollution exposure are individually known risks to human health, with amplifying adverse health effects during periods of co-exposure. This study compared co-occurring individual-level exposures to particulate matter (PM_5_, aerodynamic diameter of ≤ 5 micrometers) and heat among women in residential and agricultural settings in Guatemala. We measured personal and ambient exposure to PM_5_, temperature, and humidity among 21 female sugarcane workers in the fields and on their off days. We measured similar exposures among a group of 30 community members not involved in sugarcane work. We collected 171 personal PM_5_ measurements across 18 sampling days. The median workday personal PM_5_ concentration was 271 μg/m^3^, which was 3.6-fold higher than ambient area levels in the fields. The median personal PM_5_ concentration was 95.8 ug/m^3^ for off-work days and 83.5 ug/m^3^ for community days. The average workday individual-level temperature and humidity were 39.4°C and 82.4%, respectively, with significantly lower temperatures on off-work and community days. The women workers and community members were exposed to high levels of PM_5_ and heat in both occupational and residential settings. Research needs to consider individual-level exposures at both work and home to help tailor more effective comprehensive prevention efforts to reduce risks.

## 1. Introduction

Extreme heat events and air pollution episodes are becoming more frequent with climate change, and these exposures continue to threaten human health (1, 2). Specific populations and regions are differentially impacted by climate and environmental hazards based on geography, residence, employment, health status, sociodemographic factors, and other social determinants of health (3). For instance, workers and rural populations in tropical and sub-tropical low- and middle-income countries are likely to experience exposure to air pollution from sources such as transportation emissions, heating with solid fuels including biomass, burning waste, and agricultural practices such as burning (4). Additionally, increasing ambient temperatures pose a disproportionate threat to these same populations where seasonal heat is already very high (5–7). Moreover, these exposures likely co-exist. Another region experiencing increasing exposures is in the western U.S., where heat waves coupled with wildfires have increased in frequency and have been shown to have both individual and synergistic effects on daily cardiorespiratory hospitalizations in California (8). Understanding potential vulnerability and adaptations to these climate hazards requires individual-level concurrent exposure measurements.

Workers across various industries, specifically agriculture, wildland firefighters, construction, and transportation, are at an increased risk for exposures at work, including poor air quality and high temperatures, including in the U.S. and Latin America (9, 10). Laboring in hot conditions increases heat stress, which increases the risk of heat-related illnesses, including cramps, syncope, exhaustion, and even death from exertional heat stroke (11). In addition to heat-related illness, occupational heat stress has been associated with an increased risk of acute kidney injury and chronic kidney disease of unknown etiology (CKDu) (12, 13). Epidemics of CKDu are occurring in hot, rural agricultural communities along the Pacific Coast of Latin America, India, Sri Lanka, India, and Mexico (14–16).

Sugarcane workers in agricultural communities across Latin America have been identified as one of the occupational populations at high-risk of CKDu (17–19). In many of the countries, sugarcane fields are burned prior to harvesting cane to remove foliage and facilitate cutting. Workers re-suspend soil and/or ash while cutting and seeding cane, and they can be in the fields while neighboring fields are being burned. From our previous pilot study, we observed that sugarcane workers experience daily exposure to particulate matter (PM), soil, and ash containing silica, metals, and other potential nephrotoxins at elevated levels throughout the six-month sugarcane harvest (20). In addition to the occupational exposure to high levels of PM, living conditions in rural settings in Latin America are also conducive to increased PM exposures. The majority of households cook with solid fuels, such as wood or other biomass, which are typically burned indoors or immediately adjacent to living quarters using a three-stone open fire, making cooking an important source of household and community air pollution exposure (21). In Guatemala, women spend on average five hours per day cooking, and previous investigations reported average indoor PM_10_ (median diameter ≤10 μm) and PM_2.5_ (≤2.5 μm) concentrations of 717 μg/m^3^ and 528 μg/m^3^, respectively (22, 23). While there are several studies examining household PM exposures in this region (21, 22, 24–27), there are few studies in which personal exposure to PM has been characterized in the agricultural occupational setting, or studied in tandem with household exposure. Recent literature suggests that exposure to high temperatures in tandem with environmental exposures, such as airborne PM and its constituents, contribute to the high prevalence of CKDu observed in these countries (20). PM has the potential to penetrate the circulatory system, then reach the nephrons and be taken up by renal tubular cells, which could potentially damage renal function (28). Heat stress and dehydration might produce further adverse effects on renal function.

Given the potential for high exposure to both heat and PM among women in Latin America, an understudied population at-risk of CKDu, we conducted a pilot study to characterize co-exposure among female sugarcane workers and female community members, not currently involved in sugarcane work, living in communities next to sugarcane fields in southwest Guatemala. We prospectively evaluated exposure to PM with a 50% cut point (*d*_*50*_) of 5 μm (PM_5_) by collecting repeated personal breathing zone air samples in the sugarcane fields and during everyday activities in the household setting while simultaneously monitoring individual-level exposures to ambient temperature and humidity. We present details of our study design, data collection methods, and a characterization of occupational and residential exposures for female sugarcane workers and a comparison group of female community members. The objective of this research was to use a comprehensive approach to assess work- plus non-work exposures to both PM and heat across time in order to characterize exposures that may be contributing to acute and chronic kidney injury. To do this, we examined and compared three exposure scenarios: workday field exposures for female sugarcane workers, 2) off-work day exposures for the workers, and 3) community and household exposures for female community members. This women-focused study will advance the understanding of individual-level exposures to heat and potential airborne nephrotoxicants in the environment among female workers and contribute to development of workplace policies to protect their health. This work is part of a longitudinal study investigating occupational and non-occupational exposures and kidney health among females in Guatemala in which the community members may be the most relevant comparison group for evaluating potential exposure-related kidney effects.

## 2. Methods

### 2.1. Study Population and Design

We conducted a longitudinal study across two six-month sugarcane harvest seasons (2021–22 and 2022–23) in collaboration with a large sugarcane agribusiness in southwest Guatemala. This study was developed and conducted through a long-standing collaborative partnership with the company and its clinical staff under a memorandum of understanding with the University of Colorado. We collected repeated personal measures of PM_5_ and individual-level ambient temperature and humidity among female sugarcane workers and female members of communities within ~10 km from sugarcane fields who did not currently work in the sugarcane industry. The workers were employed as seeders and their main tasks included carrying, planting, and covering cane seeds (i.e., seeds are a cut segment of cane approximately 45 centimeters long). Workplace details have been previously described (12). The workers wear long-sleeved shirts, hats, gloves, and boots, but no respiratory protection, while in the field seeding. They work six continuous days with one day off over the weekend and their typical work shift ranges from six to eight hours.

**Figure 1.**
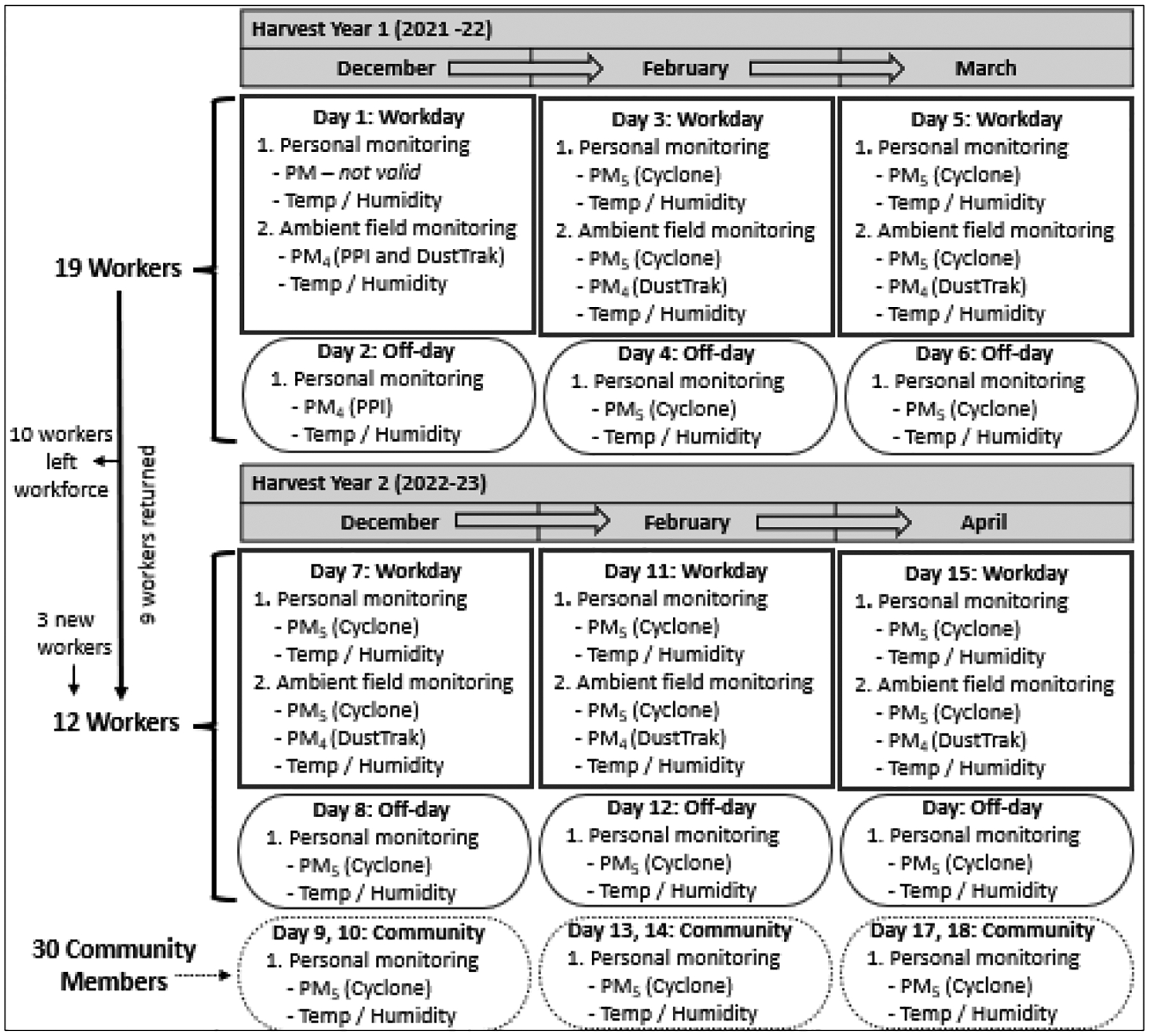
Study design and data collection details for 18 sampling days during the two harvest seasons. Workers were monitored one workday and one off-work day per month. Community members were each monitored one day per month in two different groups.

We recruited and consented all female workers (n=21) who were employed as a seeder at the agribusiness as of December 2021 or December 2022, see Figure 1. Workers either participated in one or two seasons depending on their employment duration. The harvest season starts in late November and lasts through April. The women started working the harvest one week prior to recruitment. We also recruited and consented 30 females from communities in the Departments of Escuintla and Suchitepéquez where sugarcane is the predominate crop. The community members were recruited in December 2022 and participated during the second harvest season. Inclusion criteria for community members were that they did not currently work in the sugarcane industry and that they lived in a house with either a male sugarcane cutter (26 out of 30, 87%) or lived in the same neighborhood as another participating female member (4 out of 30, 13%). All participants were ≥18 years of age and reported not being pregnant. Signed informed consent was obtained from all participants. The study was approved by the Colorado Multiple Institution Review Board (COMIRB) and in Guatemala by ZUGUEME Comité Ética Independiente.

We measured personal and ambient PM_5_ and individual- and field-level temperature and relative humidity across six sampling sessions on 18 sampling days over the two harvest seasons. The 18 sampling days comprised of the following: 1) six workdays for the workers in the sugarcane fields (three workdays per harvest), 2) six off-work days for the workers during the same week as work sampling days (three days per harvest), and 3) six sampling days for the community members during the second harvest (up to three days per community member). The worker’s off-work days and the community member sampling days were comprised of daily activities in the household and community settings. There were 21 workers (41%) and 30 community members (59%), and their ages ranged from 19 to 60 years (mean: 36, standard deviation (SD): 11). This study’s design sought to increase the understanding of total exposure to PM and heat in female sugarcane workers during their work and rest days as well as a group of community members’ exposures experienced while living in areas nearby sugarcane fields.

### 2.2. Particulate Matter Measurement

**Figure 2.**
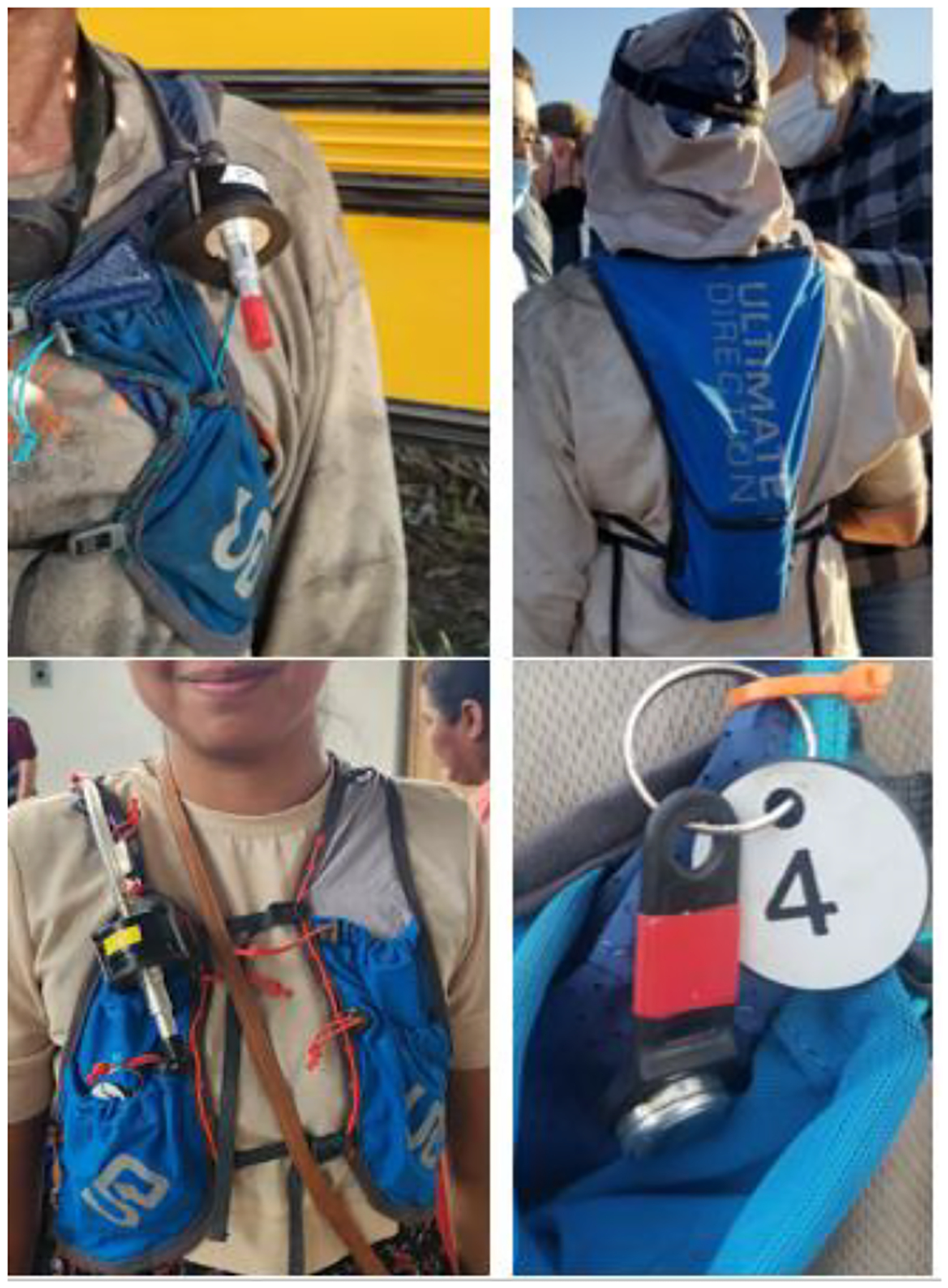
Left top picture shows the sampler set-up and vest from the front of a worker with tubing, cassette, and cyclone inlet. The right top picture shows the pump location in a pocket on the back of the vest. The left bottom picture shows the sampler set-up and vest on a community member. The right bottom is the iButton logger zip-tied to the front of the vest.

Details of our sampling approach are described in Adgate et al. (2024) (29) and are briefly summarized here. Each participant was outfitted with a size-selective sampling train. The sampling period was approximately 4 hours (half a work shift) for the workdays and approximately 6 to 8 hours for the worker’s off-work days and on the community member sampling days (~8 am to ~3 pm). Study personnel integrated air sampling systems into a lightweight, breathable vest (Ultimate Direction, Model V2, Broomfield, CO) to ensure the samplers and sampling trains (connected via tubing) were securely attached near the breathing zone of the participants (Figure 2). During workday sampling, research assistants were in the field with the workers to troubleshoot pump issues if necessary.

Two types of personal sampling inlets were deployed during this study. Initially, sampling trains were fitted with an XR5000 sampling pump attached to a SKC respirable parallel particle impactor (PPI) (SKC Inc., Eighty-Four, PA) loaded with a 37-millimeter (mm) 5.0 μm pore size polyvinyl chloride filter (PVC, SKC Model 225-5-37). However, our first study workday samples (December 2021) were not valid due to overloading of the impaction plates. Given the large contribution of coarse particles to the exposure, we switched to a SKC aluminum Cyclone sampler (Model 225-01-02, Eighty-Four, PA) to address the overloading issue. By design, cyclone samplers use centrifugal force to segregate larger particles (i.e., non-respirable) into a small receptacle that is away from the filter instead of impaction plates directly downstream of the inlet that are more susceptible to overloading from large non-respirable particles in this environment. These samplers were also loaded with a 37-mm pore size of 5.0 μm polyvinyl chloride filter (PVC, SKC Model 225-5-37). The cyclone samplers were used for all subsequent work, off-work, and community measurements. To protect against overloading the cyclone samplers, we reduced the duration of sampling to 4 hours for each workday. For the first harvest, workers were randomly assigned to either the first 4-hours the work shift (morning) or after a 4-hour delay (afternoon). During the second harvest we only sampled during the mornings on workdays due to shortened shift durations that could not accommodate a second 4-hour measurement. Sampling trains were calibrated to 4 L/min for PPI samplers and 2 L/min for cyclone samplers. While the PPI samplers collected respirable PM (i.e., those particles with a 50% cut point of 4 μm) the inlet efficiency of these cyclone samplers at 2 L/min has been reported to be 50% for particles 5 μm in diameter (PM_5_).

Full shift (~ 8 hour) ambient particulate matter samples were collected during workdays with a stationary PPI measuring PM_4_ (December 2021) and cyclone measuring PM_5_ (February 2022-April 2023). These samples were co-located with a DustTrak DRX Aerosol Monitor (TSI, Shoreview MN) measuring real-time PM_4_. Ambient samplers and the DustTrak monitor were placed on a table approximately 1 meter off the ground in the field near the workers for the duration of the work shift.

### 2.3. Heat Exposure Monitoring

We collected individual-level exposure to heat using a wireless iButton sensor (Hygrochron^™^, model DS1923, Maxim Integrated, San Jose, CA) affixed to a key fob attached to the front of the marathon vest (Figure 2) to avoid direct sunlight, which can cause higher temperatures, and from getting covered in dust (30). The iButtons measured each participant’s individual-level ambient temperature and relative humidity at 2-min intervals throughout the sampling period on work, off-work, and community sampling days. The iButtons have a range of −20 to 85 °C at a 0.5 °C resolution with a temperature precision of ±0.5 °C and timing precision of ±2 min per month. iButton thermometers were factory-calibrated using NIST standards (31).

We calculated individual-level average and maximum temperature and humidity measures during the entire work shift (~8 hours) and during the entire sampling period for off-work days and community days (~ 6 to 8 hours). We also calculated heat index using the National Oceanic and Atmospheric Administration (NOAA) Rothfusz equation (32). Heat index was calculated at every 2-min interval based on the temperature and relative humidity and then heat index was summarized as daily average and maximum values. Heat index is a combination of temperature and humidity and is often used for heat advisories by the National Weather Service and by the U.S. Occupational Safety and Health Administration (OSHA) for assessing risk of heat-related illnesses. We compared the iButton heat index measures with OSHA’s established risk categories in degrees Fahrenheit (33).

In addition to individual-level heat monitoring, we collected field-level meteorological data during the entire work shift only on the study workdays. A Wet Bulb Globe Temperature (WBGT) meter (Kestrel 5400, Boothwyn, PA) was placed in the same field where the participants were working to estimate field-level ambient temperature, relative humidity, and wind speed.

### 2.4. Lab Analyses

The PVC filters were weighed before and after each sampling event at Colorado State University using a Mettler MT5 Balance (Mettler-Toledo, Inc., Columbus, OH) to determine collected sample mass. Prior to gravimetric analysis, filters were desiccated for at least 24 hours and static neutralized with a U-electrode (Mettler-Toledo, Inc.). All gravimetric analysis was performed in a temperature and humidity-controlled environment. Time-weighted average concentrations were determined by dividing the mass on each filter by the volume of air sampled. Laboratory and field blank filters were used to correct for measurement error. PM results are presented in μg/m^3^.

### 2.5. Statistical Analysis

We characterized distributions of PM levels and heat metrics using descriptive statistics. The distributions of the workday concentrations, off-work concentrations, and community concentrations were skewed; thus, comparisons between the days were assessed using nonparametric Mann Whitney Wilcoxon and Kruskal-Wallis tests. We compared PM 4-hour sampling periods in the morning (n=11) and afternoon (n=11) from the two workdays during the first harvest (February and March 2022) and found no significant difference between the means of the morning and the afternoon sampling periods (p=0.74), so we combined all 4-hour workday sampling periods (Supplemental Table S1).

We examined correlations between the individual-level heat monitoring metrics (average and maximum for temperature, humidity, and heat index) and personal PM concentrations by work, off-work, and community sampling days. For this analysis, we calculated the individual-level heat metrics to coincide with the respective individual air monitoring duration. For example, on workdays, if the air measurement was for 4-hours in the morning, we calculated individual average and maximum heat indices for the same 4-hour period. All analyses were conducted in SAS version 9.4 (Cary, NC).

## 3. Results

### 3.1. Particulate Matter Exposure

We obtained 171 personal PM_5_ measurements from the 51 participants (96% of 178 attempted). Four samples were not valid during off-work days during Year 1 and three samples were not valid during off-work days in Year 2. Of the 171 samples, 51 were collected across five workdays, 62 samples were collected across six off-work days for the workers, and 58 samples were collected across six community member days.

**Figure 3.**
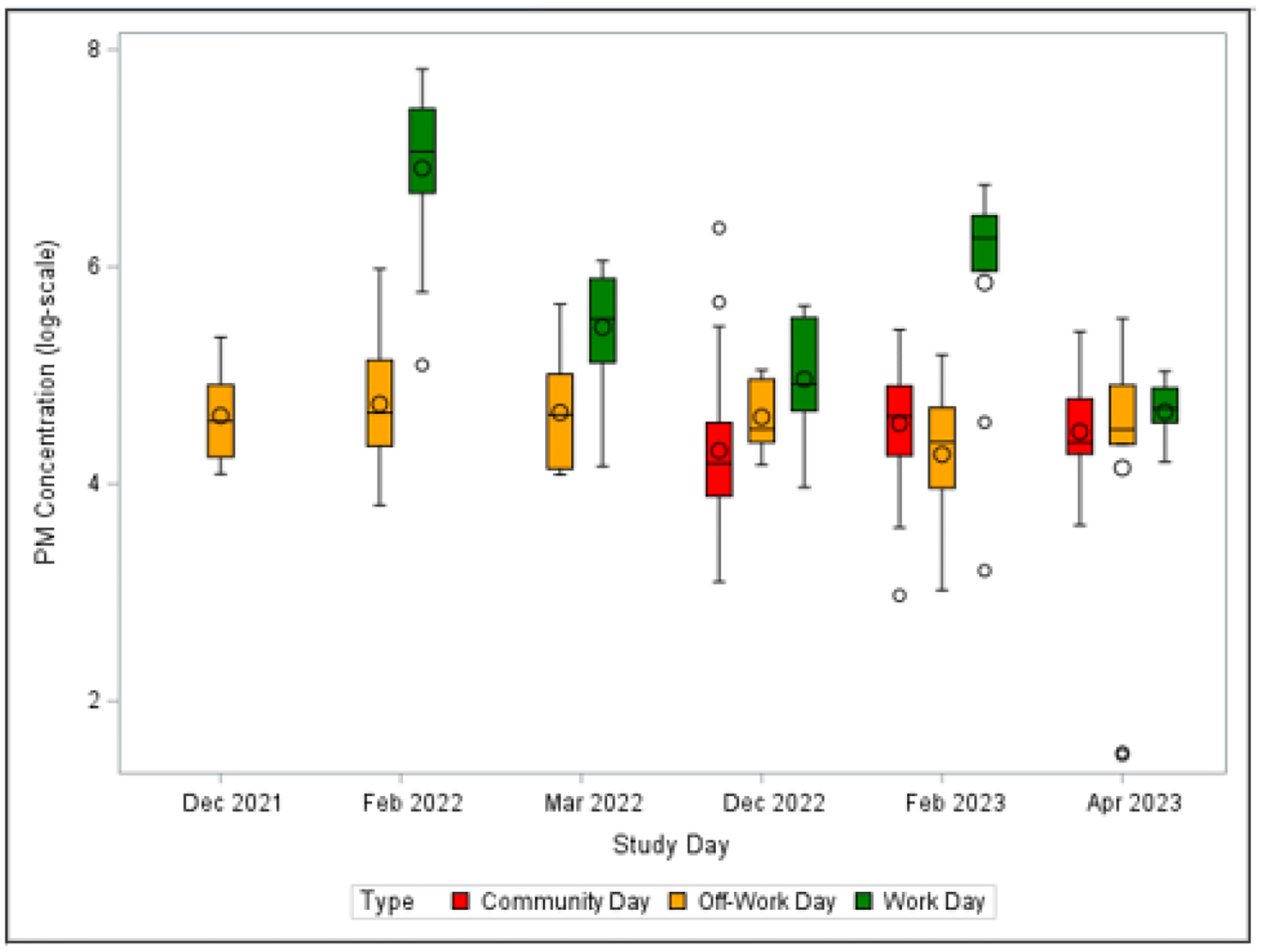
Box plots of log-transformed personal particulate matter (PM_5_) measurements by sampling month and day type (community, off-work, and work) across two study harvest seasons. Workday samples were not valid in Dec 2021. Community days were only conducted during the second harvest.

Personal breathing zone concentrations of PM_5_ during each sampling session are shown in Table 1. The distribution of mass concentrations by sampling month and day type are presented in Figure 3, and the distribution by sample year and day type are presented in Supplement Figure S1. Median personal PM_5_ concentrations were significantly higher on workdays compared to off-work days and community days despite having a shorter sampling period (271.7, 95.8, and 83.5 μg/m^3^, respectively, *χ*^*2*^ = 44.1, *p* = < .001, *df* = 2). Workday PM_5_ concentrations ranged from 24.5 to 2492 μg/m^3^, with a median value of 271.7 μg/m^3^. There was a significant difference between workday PM_5_ concentrations (χ^2^= 25.3, *p* = < .001, *df* = 4). February 2022 and February 2023 had higher median workday concentrations (1167 and 526.2 μg/m^3^, respectively) compared to March 2022 (249.4 μg/m^3^), December 2022 (134.0 μg/m^3^), and April 2023 (110.3 μg/m^3^). It was noted that the study field on the workday in February 2022 had been recently plowed and was dry, and the field on the workday in March 2022 had recently been irrigated. Personal PM_5_ concentrations for the workers on their off-work days ranged from 4.5 to 396.3 μg/m^3^, with a median value of 95.8 μg/m^3^. The median concentration for community member sampling days was slightly lower at 83.5 μg/m^3^ with a range from 19.5 to 576.4 μg/m^3^.

The time weighted average ambient PM_5_ measurements on the workdays ranged from 63.59 to 195.3 μg/m^3^ (Table 2). The mean ambient PM_5_ level was 3.6-fold lower than the mean personal PM_5_ level, with lower levels on four of the five workdays. The mean PM_4_ concentrations for ambient DustTrak samples ranged from 55–168 μg/m^3^.

### 3.2. Heat Exposure

**Figure 4.**
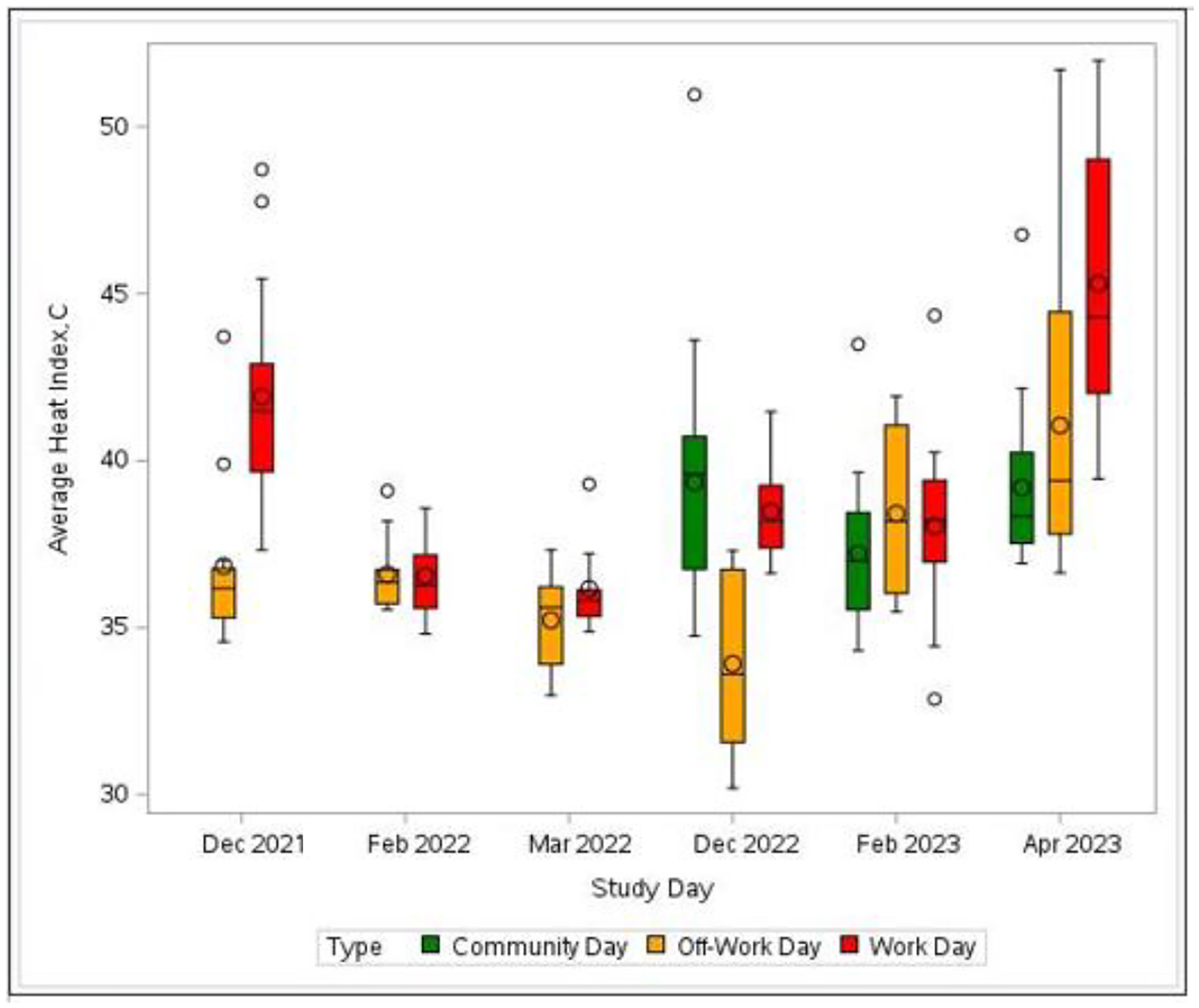
Box plots of average heat index levels by sampling month and day type (community, off-work, work) across two study harvest seasons. Community days were only conducted during the second harvest.

For all study days, the means of the individual-level average temperatures ranged from 30.8°C to 35.3°C, and the means of the individual maximum temperatures ranged from 36.4°C to 43.3°C (Table 3). The average heat index and maximum heat index were 39.4°C and 53.6°C (102.9°F and 128.5°F, respectively) for workdays, and 37.0°C and 47.8°C (98.6°F and 118.0°F, respectively) for the off-work days. For the community days, the average heat index was 38.6°C (101.4°F), and the maximum heat index was 51.3°C (124.2°F). The distribution of heat index levels by sampling month and day type are presented in Figure 4. Box plot figures of the distributions for temperature and humidity are presented in the Supplemental Figures S2 and S3. All individual-level heat exposure indices were significantly different between the types of days (average heat index: χ^2^ = 13.7, *p* = .001; maximum heat index: χ^2^ = 13.7, *p* = .001; maximum temperature: χ^2^ = 45.4, *p* <.001; average temperature: χ^2^ = 13.6, *p* = .001; maximum humidity: χ^2^ = 17.4, *p* < .001; and average humidity: χ^2^ = 20.7, *p* < .001), Table 3. Workdays had significantly higher heat indices compared to the off-days and community days, except for average humidity, which was higher on the community days. Furthermore, average individual-level temperatures were higher than the field-level temperatures on all five workdays, with two of the workdays having average individual-level temperatures 2.4 °C higher than the field temperatures. Individual-level humidity measures were higher than field humidity measures on two of the five workdays.

When comparing the observed individual maximum heat index levels to the U.S. OSHA heat index-based risk levels, 13 of the 18 sampling days (72%) were categorized as very high to extreme risk (Supplemental Table S2). All of the community days (n=6/6), one-third of the off-work days for workers (n=2/6) and almost all of the workdays (n=5/6) categorized as very high to extreme risk days.

Moderate negative correlations were found between both individual-level average and maximum humidity and PM_5_ on workdays (*r* = −.54, *r* = −.52 respectively, *p* < .001 for both). Additionally, moderate negative correlations were found for individual-level average and maximum heat index and PM_5_ on workdays (*r* = −.46, *r* = −.67, respectively, *p* < .001 for both). Therefore, as humidity and heat index increased, PM_5_ concentrations were observed to decrease on workdays. On the off-work days, there was a weak negative correlation between individual-level maximum temperature and PM_5_ (*r* = −.27, *p* = .04). No correlations were observed between PM_5_ and any of the heat indices on the community sampling days. We observed fewer correlations on the off-work and community days which may be due to varied tasks throughout the day, both indoors and outdoors.

## 4. Discussion

This research found that women in rural Guatemala are exposed to high levels of airborne PM_5_ and high heat index levels at the workplace as well as in the home environment when sugar harvesting is performed in the dry season. Workday personal PM_5_ measurements were significantly higher than personal PM_5_ measurements on the off-work days and community days. In addition, personal exposure measurements of PM_5_ and individual-level temperature were higher than ambient PM_5_ and field-level temperatures on workdays. Future occupational studies should be cautious in the interpretation of area measurements as estimates of personal exposure when examining exposure-response relationships. This study demonstrates the need to measure exposures in both occupational and non-occupational environments to better understand an individuals’ exposure risks in agricultural communities.

The workday personal PM_5_ measurements ranged from 24.5 to 2492 μg/m^3^, with a median value of 271.7 μg/m^3^. We observed that on workdays none of the workers were exposed to levels of PM exceeding the OSHA permissible exposure limit (PEL) for respirable particulates not otherwise regulated at 5 mg/m^3^ (OSHA, Code of Federal Regulations, Title 29). However, using this PEL is not relevant, especially since this guidance applies to particles that are inert or considered of “low toxicity,”(34) which reduces its applicability for this study given that amorphous silica and metals are known to be present (20).

We observed highly variable personal PM_5_ measurements between workdays despite worker seeding job tasks being consistent across workdays. PM_5_ concentration on the two February workdays during both harvest seasons were significantly higher than the three other workdays. One possible explanation for the variation is soil moisture differences between the sampling periods. Notably, we observed that the field where the workers were seeding in February 2022 was recently plowed and dry, while in March 2022, the study field had been recently irrigated. During seeding, which is usually performed after land preparation activities, particles are resuspended by wind and worker practices due to the soil structure loss during planting seed cane segments (35). A recent study has shown that PM_2.5_ variability is linked to soil moisture in which low soil moisture contributes to elevated PM levels (36). Another possible explanation for PM measurement variability is the sample location proximity to nearby dirt roads, which serve as byways for large trucks and heavy equipment. Large trucks on these roads generate substantial amounts of airborne dust. On the April 2023 workday, personal PM concentrations were relatively low and lower than the ambient PM concentration, however, personal heat index levels were high. It was noted by field research staff that several workers spent more time resting in the shade that day, potentially limiting personal PM exposure. Understanding the work behaviors and other factors that influence these variations in occupational exposure can help inform preventive measures to reduce PM and heat exposure.

For purposes of comparison, we researched but were unable to find other published occupational air pollution exposure assessment studies among manual field workers in similar field and climatic conditions. Several studies in the region have measured household air pollution in relation to cooking practices, mainly PM_2.5_ in woodstove using homes. Indoor PM_2.5_ concentrations in Guatemala are often higher (37) than the World Health Organization (WHO) air quality guideline for PM, which currently stands at 10 μg/m^3^ as an annual mean and 25 μg/m^3^ as a 24-h mean (38). In a study among 49 rural Honduran women, mean 24-hour personal PM_2.5_ concentrations, using methods similar to ours, were 60.2 ± 25.7 μg/m^3^ (24). Another study in Honduras observed that traditional stove users in Honduras had mean personal 24-hour PM_2.5_ concentrations of 126 μg/m^3^ (39). Although our area and personal samples measured a slightly higher cut point (PM_5_), our measured concentrations were well above the WHO health-based guidelines. Another occupational air pollution exposure, wildfire-related PM, may be worthy of comparison. Wildfire smoke is a complex mixture of gases and particles from burning vegetation, and the smoke is made up of potentially toxic compounds. Our PM_5_ workday concentrations were higher than the Oregon OSHA wildfire smoke standard for PM_2.5_ at 35.5 μg/m^3^ (Air Quality Index, AQI, value of 101), and California’s standard at 55.5 μg/m^3^ (AQI value of 151)(40, 41).

Our data demonstrate that workers both at work and off-work, and community members, were exposed to high temperatures and humidity. Moreover, we observed that heat exposure was significantly higher on workdays compared to the off-work days with average individual maximum temperatures as high as 43.3°C. OSHA has identified levels of risk based on the heat index, ranging from low risk to extreme risk, and all of our sampled days fall into the high or very high extreme categories based on the individual heat index maximum levels (33). García-Trabanino et al. (2015) measured field temperature and humidity during similar work shift hours on six days in Nicaragua (42). They observed mean heat index ranging from 98 –111 °F. The mean average heat index for our current study ranged from 97.1 to 113.5°F on the workdays. Heat indices meeting or exceeding 103°F can lead to dangerous heat disorders, and other adverse health outcomes including kidney injury, with prolonged exposure and/or physical activity in the heat. In addition, the workers wear protective clothing and work in full sunshine during their work shift. In these scenarios, OSHA recommends providing access to adequate potable water, acclimatizing workers, and modifying work rest cycles based on established American Conference of Governmental Industrial Hygienists (ACGIH) guidelines (33, 43). ACGIH guidelines provide recommendations on work/rest schedules according to WBGT and workload categories (referred to as ‘metabolic work rates’) (44). A modified work/rest cycle could potentially reduce PM exposure as noted above. The agribusiness where the workers were employed has already implemented a water, electrolytes, rest, and shade program to reduce heat stress among their workers. Details of this program have been previously published (45).

Limitations of the study include the relatively small number of participants in a targeted geographic area during the dry season, thus limiting generalizability of our findings. We also used convenience sampling for recruitment of the community women, consequently their exposures may not be representative of the population at large. Different sampling equipment was used in December 2021 (PPI) compared to the subsequent sessions (cyclone) although off-work PPI sampler measurements were not significantly different than subsequent off-work cyclone-based measurements. Due to the field conditions, only 4 hours of sampling was conducted for each worker on their workdays; however, we were able to show that the morning and afternoon 4-hour periods were similar, consistent with the observation that workers conducted the same tasks during the entire 8-hour work shift. Variation in sample duration due to varying schedules and conditions may be a source of unrecognized bias. For example, workday sample duration was consistent except for March 2022 when afternoon rain cut our sampling time short, which may have resulted in lower PM concentrations. We acknowledge that sugarcane fieldwork involves a diverse range of tasks and field conditions making it difficult to generalize findings from this study regarding potentially harmful PM exposures across an entire harvest season or to other agricultural environments.

This study included personal air and heat monitoring conducted during work and around the household among two female populations at-risk for disproportionate exposure to climate hazards. Females are often considered to undertake less hazardous work compared to men; however, this study suggests that women’s’ exposure to PM and heat extremes should not be discounted in studies looking for exposure-related adverse health outcomes. We also observed that workers and community members had similar residential PM and heat exposure levels, suggesting that nearby community member residences are good proxies for exposures of female workers when they are off the job. By using personal exposure sampling methods, we can more accurately characterize exposures in the workplace and during performance of everyday household activities, and, in turn, more precisely assess exposure impacts on kidney health, which we will do in future studies. It is important to note that heat stress has been shown to exacerbate the effects of airborne pollutants, causing a multiplier negative effect on health (46–48). Increasing our understanding of daily exposures to extreme heat and poor air quality can provide greater insight on the health effects of these co-existing exposures as well as other adverse effects including worker productivity and the effects on livelihoods in low socio-economic communities.

## 5. Conclusions

We monitored personal PM and heat exposures at work and at home and demonstrated elevated levels for women agricultural workers compared to area sampling levels and air quality benchmarks. We also demonstrated that the residential PM and heat exposures for community members are elevated compared to standard guidelines. These findings show that it’s necessary to address both work and community air pollution, and heat exposures among women in future research and in the development of preventative strategies. In addition, our findings emphasize the need to estimate exposure using personal sampling measurements to better characterize and understand the impacts of combined exposures on populations at-risk to adverse health outcomes, such as CKDu. Further studies considering the synergistic health effects of co-occurring heat and PM exposures are warranted. Research that considers individual exposures at both work and home can help tailor more effective comprehensive prevention efforts to reduce risks, thereby preventing disease, for workers, their families, and their communities.

## Supplementary Material

Supplementary

## Figures and Tables

**Table 1.** Summary statistics for 171 personal PM_5_ (μg/m^3^) from 21 female sugarcane workers and 30 female community members at six sampling sessions across two harvest seasons.

	Overall			Harvest 2021–2022						Harvest 2022–2023								
Month				December		February		March		December			February			April		
Day Type	Work	Off-work*	Comm	Work	Off-work	Work**	Off-work	Work**	Off-work	Work	Off-work	Comm	Work	Off-work	Comm	Work	Off-work	Comm
Valid samples ^A^	51	62	58	0	13	13 ^B^	13	9 ^C^	7	9 ^D^	8	24	10 ^D^	10	19	10 ^D^	11	15
Sample duration, minutes, mean (SD)	233 (23)	437 (59)	372 (31)	NA	468 (19)	240 (0)	480 (0)	199 (41)	538 (9)	240 (0)	360 (6)	347 (18)	240 (0)	395 (9.5)	406 (21)	240 (0)	382 (13)	369 (14)
Minimum	24.5	4.5	19.5	NA	59.7	163.1	44.8	64.1	59.4	52.9	65.1	22.19	24.5	20.5	19.5	66.8	4.5	37.4
P25	124.4	73.6	61.6	NA	70.0	796.3	77.3	167	62.7	106.8	79.9	48.8	387.8	52.6	70.8	96	78.6	71.8
Median	271.7	95.8	83.5	NA	97.4	1167	105.0	249.4	103.3	137.0	90.5	65.9	526.2	80.3	102.1	110.3	90.2	79.7
P75	652.0	141.1	119.4	NA	136.2	1724	170.8	362.3	149.9	252.2	143.1	96.0	646.9	110.4	134.6	131.7	135.7	119.4
Maximum	2492	396.3	576.4	NA	211.0	2492	396.3	426.8	286.1	281.8	156.2	576.4	857.0	178.9	226.4	154.2	250.1	221.9

Comm, community sampling day. SD, Standard Deviation. P25 and P75 denote the 25^th^ & 75^th^ percentiles.

* Off-work samples represent household activities (i.e., not in the sugarcane field).

** Study fields were recently plowed in Feb 2022 and recently irrigated in March 2022.

A Samples were collected using Cyclone samplers, except in December 2021, work samples were collected using parallel particle impactor (PPI) samplers.

B 6 (46%) samples were collected during afternoon 4 hours of work shift (vs. morning 4 hours).

C 5 (56%) samples were collected during afternoon 4 hours of work shift (vs. morning 4 hours).

D All morning 4-hour samples.

**Table 2.** Summary statistics for full work shift (~8 hours) ambient PM_5_ measurements and field weather indices collected in the same field as the personal samples for female sugarcane workers across two harvest seasons.

	December 2021	February 2022	March 2022	December 2022	February 2023	April 2023
**Ambient PM** ^A^**, μg/m**^**3**^						
PM_5_	89.4	116.4	153.4	63.6	84.1	195.3
Duration, min	491	571	428	204	402	444
**DustTrak, μg/m** ^**3**^ **, mean (min-max)**						
PM_4_	102 (16–2430)	106 (19–1770)	158 (41–4060)	55 (9–4490)	168 (27–2700)	95 (47–1170)
**Field Weather Indices, mean (standard deviation)**						
Temperature, °C	33.0 (2.5)	31.7 (2.1)	28.4 (2.8)	29.7 (1.9)	N/A	33.7 (1.9)
Relative Humidity, %	52.1 (7.0)	51.5 (7.3)	75.3 (19.9)	65.8 (7.25)	N/A	45.0 (8.1)

A A parallel particle impactor (PPI) sampler was used to collect the December 2021 ambient measurement at PM_4_. A cyclone sampler was used to collect all other ambient measurements at PM_5_. Field weather indices for February 2023 were missing due to technical issues with the Wet Bulb Globe Temperature meter.

**Table 3.** Summary statistics for personal heat exposure measurements from 21 female sugarcane workers and 30 female community members across the two harvest seasons.

	Overall			Harvest 2021–2022						Harvest 2022–2023								
Month				December		February		March		December			February			April		
Day Type	Work	Off-work ^A^	Comm	Work	Off-work	Work	Off-work	Work	Off-work	Work	Off-work	Comm	Work	Off-work	Comm	Work	Off-work	Comm
N	65	62	57	14	13	13	13	9	7	9	8	24	10	10	19	10	11	15
Average Humidity, %	58.4 (9.9)*	54.9 (6.6)	61.8 (7.4)	60.2 (7.7)	54.0 (4.8)	45.7 (2.9)	47.2 (3.4)	67.5 (6.2)	55.8 (4.1)	63.7 (4.4)	63.4 (2.9)	65.1 (7.1)	59.5 (9.3)	54.9 (5.7)	54.8 (4.3)	58.1 (10.2)	58.1 (5.7)	65.1 (4.5)
Maximum Humidity, %	82.4 (10.6)*	74.2 (10.6)	78.9 (7.7)	87.2 (8.4)	74.2 (5.7)	68.6 (7.3)	60.3 (10.6)	94.5 (2.9)	75.3 (5.5)	86.4 (5.2)	85.0 (1.6)	80.2 (9.0)	79.4 (8.1)	79.9 (9.4)	75.9 (6.6)	82.1 (6.6)	77.1 (5.2)	80.6 (5.6)
Average Temperature, °C	33.0 (1.6)*	32.6 (1.5)	32.2 (0.9)	33.8 (1.1)	32.6 (0.7)	33.8 (0.9)	33.5 (0.8)	30.8 (0.5)	31.4 (1.0)	32.1 (0.7)	30.1 (1.3)	32.0 (0.6)	32.2 (1.3)	33.1 (1.0)	32.6 (1.0)	35.3 (0.6)	33.5 (0.9)	32.1 (0.9)
Maximum Temperature, °C	39.4 (3.1)*	36.5 (1.6)	36.6 (2.9)	43.3 (3.0)	36.8 (1.7)	37.9 (1.4)	37.1 (1.3)	36.4 (1.7)	36.7 (2.6)	38.7 (3.2)	34.7 (0.9)	36.1 (2.0)	38.7 (1.6)	36.5 (0.8)	37.7 (4.3)	39.6 (0.9)	36.7 (1.4)	36.0 (1.2)
Average Heat Index, °C	39.5 (4.2)*	37.2 (3.4)	38.6 (3.1)	41.9 (3.4)	36.8 (2.4)	36.6 (1.2)	36.6 (1.1)	36.2 (1.3)	35.2 (1.5)	51.4 (9.1)	33.9 (2.8)	39.3 (3.6)	38 (3.3)	38.4 (2.5)	37.2 (2.2)	45.3 (4.2)	41.1 (4.5)	39.2 (2.6)
Maximum Heat Index, °C	53.7 (11.2)*	47.8 (7.4)	51.6 (7.7)	63.2 (14.8)	45.3 (5.3)	42.0 (2.2)	43.5 (3.3)	50.2 (6.5)	44.4 (5.0)	54.9 (8.3)	44.6 (6.1)	53.7 (8.9)	53.4 (6.6)	54.5 (4.6)	50.2 (7.7)	58.0 (7.2)	54.3 (9.5)	49.9 (4.6)

Mean (SD, Standard Deviation) are presented. Comm, community sampling day.

A Off-work samples represent daily household activities (i.e., not in the sugarcane field) from workers.

* Denotes a *p*-value ≤ 0.05 comparing overall work, off-work, and community values using Kruskal–Wallis test.

## Data Availability

The data presented in this study are available on request from the corresponding author.
