# Supplementary material for: Concurrent Particulate Matter and Heat Exposure in Working and Non-working Women in Rural Guatemala
Source: Atmosphere (Basel). Author manuscript; Available in PMC 2025 Sep 20. (PMC12448059; doi:10.3390/atmos15101175)
Supplement: Supplementary [file NIHMS2105148-supplement-Supplementary.pdf]

atmosphere

MDPI

Supplementary Materials: Concurrent Particulate Matter and Heat Exposure in Working and Non-working Women in Rural Guatemala

Jaime Butler-Dawson [1,2,*], Grant Erlandson [3], Diana Jaramillo [1,2], Laura Calvimontes [3], Daniel Pilloni [4], James Seidel [3], Colton Castro [3], Karely Villarreal Hernandez [1], Lyndsay Krisher [1,2], Stephen Brindley [1,2], Miranda Dally [1,2], Alex Cruz [4], Katherine A. James [1,2], Lee S. Newman [1,2,5,6], Joshua W. Schaeffer [3] and John L. Adgate [1,2]

Table S1. Summary statistics for ~4-hour personal particulate matter measurements (PM_5 in μg/m^3) from 14 female sugarcane workers for two workdays stratified by the morning (AM) or afternoon (PM) collection times for the sampling campaigns during the first study harvest season.

~4-hour PM_5 concentrations	February 2022 Workday		March 2022 Workday	
	**AM**	**PM**	**AM**	**PM**
N samples	7	6	4	5
Duration, min, mean (SD)	240 (0)	240 (0)	240 (0)	166 (15)
Min	163.1	319.6	342.1	64.12
P25	774.6	981.9	352.2	155.4
Median	963.8	1608	372.7	167.0
P75	1313	1958	404.9	223.1
Max	1724	2492	426.8	249.4

Wilcoxon signed rank test for AM samples vs. PM samples, p=0.74.

Table S2. Percentage of days meeting criteria for U.S. Occupational Safety and Health Administration heat index-based risk levels during the 18 sampling days across two harvest seasons.

	**Mean of Individual Average Heat Index**	**Mean of Individual Maximum Heat Index**
**Risk Level Experienced**		
Low, <91 °F (<32.7 °C)	0%	0%
Moderate, 91°F to 103 °F (32.7 °C - 39.4 °C)	72%	0%
High, 103°F to 115 °F (39.4 °C - 46.1 °C)	28%	28%
Very High to Extreme, >115 °F (>46.1 °C)	0%	72%

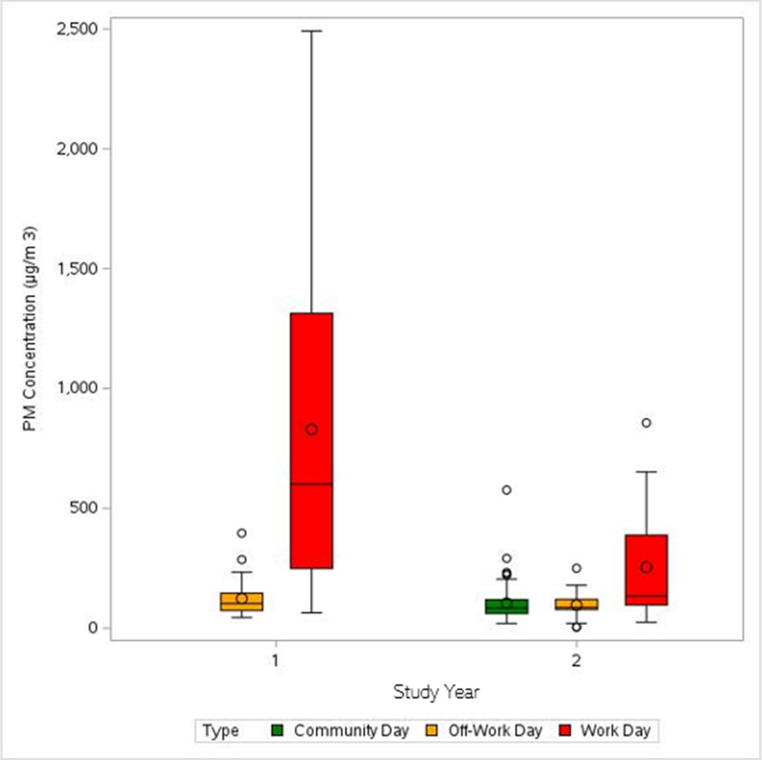

Figure S1. Box plots of personal PM_5 measurements (µg/m^3), on workdays, off-work days, and community days (Year 2 only) by study harvest year.

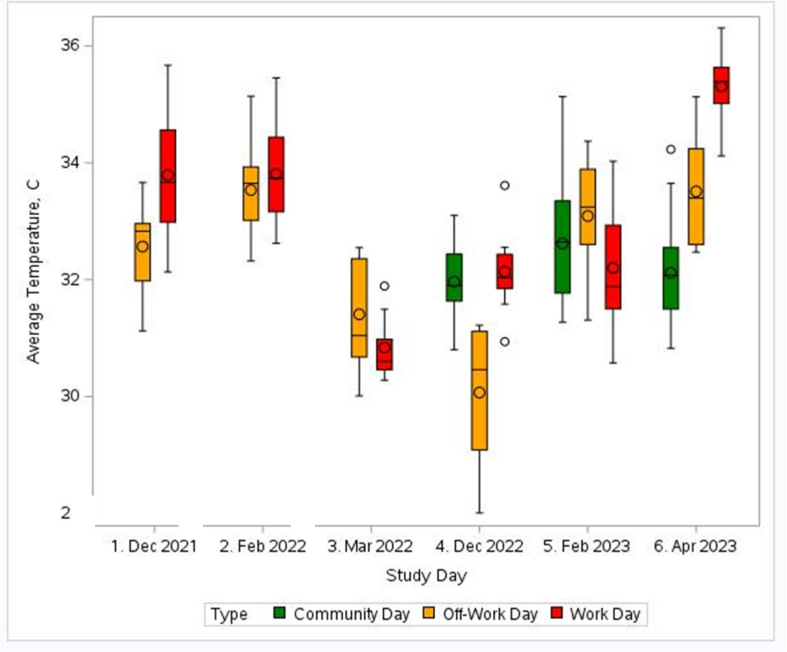

Figure S2. Box plots of average temperature by sampling month, and day type (community, off-work, work) across two study harvest seasons. Community sampling days occurred only during the 2nd harvest.

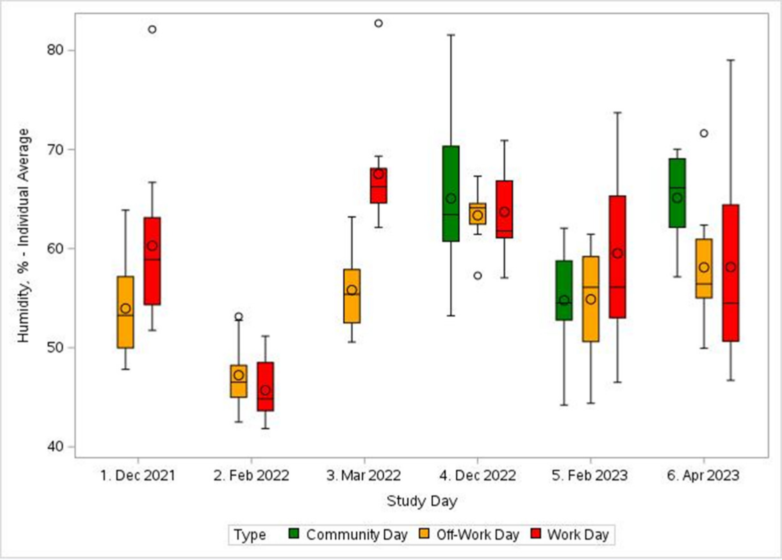

Figure S3. Box plots of average relative humidity by sampling, month and day type (community, off-work, work) across two study harvest seasons. Community sampling days occurred only during the 2nd harvest.
